# Supplementary material for: HPV self-sampling for cervical cancer screening during wartime: a pilot implementation study from a conflict-affected region of Ukraine
Source: BMJ Glob Health. 2026 Apr 28;11(4):e023478. doi: 10.1136/bmjgh-2026-023478 (PMC13141220; doi:10.1136/bmjgh-2026-023478)
Supplement: online supplemental file 1 [file bmjgh-11-4-s001.docx]

### BMJ Global Health Author Reflexivity Statement

Adapted from Morton, B., Vercueil, A., Masekela, R., Heinz, E., Reimer, L., Saleh, S., Kalinga, C., Seekles, M., Biccard, B., Chakaya, J., Abimbola, S., Obasi, A. and Oriyo, N. (2022), Consensus statement on measures to promote equitable authorship in the publication of research from international partnerships. Anaesthesia, 77: 264-276. <https://doi.org/10.1111/anae.15597>

| **Study conceptualisation** | |
| --- | --- |
| 1. How does this study address local research and policy priorities? | The study addresses an urgent local public health priority in Ukraine: maintaining access to cervical cancer screening during active war, when routine health services are disrupted. The research focuses on feasible, safe, and scalable HPV self-sampling strategies adapted to wartime conditions, aligned with national cervical cancer prevention goals and immediate clinical needs identified by Ukrainian healthcare providers. |
| 1. How were local researchers involved in study design? | Local Ukrainian clinicians, researchers, and programme implementers were involved from the outset in defining the research question, objectives, and study design. Study priorities were driven by local clinical realities, including security constraints, population displacement, and limitations in follow-up capacity. Ukrainian partners led decisions on recruitment strategies, operational workflows, and follow-up pathways to ensure contextual appropriateness and participant safety. |
| **Research management** | |
| 1. How has funding been used to support the local research team(s)? | Study funding was used to support local implementation activities in Ukraine, including personnel involved in participant recruitment, sample collection, clinical follow-up, and coordination with healthcare facilities. Resources were allocated to ensure continuity of local screening activities, training, and secure data handling under wartime conditions. |
| **Data acquisition and analysis** | |
| 1. How are research staff who conducted data collection acknowledged? | Research staff responsible for field implementation, recruitment, sample handling, and clinical follow-up in Ukraine are included as co-authors or acknowledged contributors, reflecting their substantive role in data generation and study execution. |
| 1. How have members of the research partnership been provided with access to study data? | All partners had access to study data through a secure digital platform hosted by the Swedish institution, with controlled access levels to ensure data protection. Ukrainian investigators had full access to locally generate data and were involved throughout data cleaning and verification. |
| 1. How were data used to develop analytical skills within the partnership? | The primary focus of the study was on implementation and service delivery rather than advanced statistical analysis. Data were used collaboratively to support descriptive analyses, operational decision-making, and contextual interpretation, with analytical decisions discussed within the partnership. |
| **Data interpretation** | |
| 1. How have research partners collaborated in interpreting study data? | Interpretation of results was led by senior authors in close collaboration with Ukrainian investigators. Findings were discussed iteratively within the full author group to ensure contextual accuracy, appropriate framing of results, and balanced interpretation relevant to conflict-affected settings. |
| **Drafting and revising for intellectual content** | |
| 1. How were research partners supported to develop writing skills? | The manuscript was drafted by the senior author, with all co-authors critically reviewing, revising, and contributing to the intellectual content through multiple rounds of feedback. Writing and revision were conducted as a collaborative process, ensuring accuracy, clarity, and incorporation of local clinical and implementation perspectives. |
| 1. How will research products be shared to address local needs? | Study findings will be shared with local healthcare providers, programme implementers, and relevant stakeholders in Ukraine to inform ongoing screening activities and policy discussions. Results are intended to support practical decision-making in similar conflict-affected and humanitarian contexts. |
| **Authorship** | |
| 1. How is the leadership, contribution and ownership of this work by LMIC researchers recognised within the authorship? | Authorship reflects substantive contributions across study conception, implementation, analysis, and manuscript preparation. Ukrainian authors are represented in leading authorship positions, including primary corresponding authorship, reflecting local ownership, leadership, and responsibility for the research. |
| 1. How have early career researchers across the partnership been included within the authorship team? | The authorship team primarily comprises senior clinicians, programme implementers, and experienced researchers directly responsible for study design, implementation, analysis, and reporting. Due to the urgent and operational nature of the study conducted during active war, early career researchers were not formally included at this stage. As the programme scales and stabilises, early career researchers are planned to be recruited and integrated into future research activities. |
| 1. How has gender balance been addressed within the authorship? | Gender balance within the authorship reflects the composition of the clinical and research teams involved in the study, with representation across senior and leadership roles. |
| **Training** | |
| 1. How has the project contributed to training of LMIC researchers? | The project contributed to training in HPV screening implementation, data management, quality assurance, and research reporting for Ukrainian researchers and healthcare professionals, strengthening local capacity under challenging conditions. |
| **Infrastructure** | |
| 1. How has the project contributed to improvements in local infrastructure? | The study supported the use of secure digital infrastructure for data management and result reporting, laboratory quality assurance systems, and standardised operational procedures that can be sustained beyond the study period. |
| **Governance** | |
| 1. What safeguarding procedures were used to protect local study participants and researchers? | Safeguarding procedures were adapted to wartime conditions and included ethical approval, secure data handling, minimisation of participant burden, and flexible operational protocols prioritising safety. Decisions regarding recruitment and follow-up were led locally to respond to evolving security risks and healthcare constraints. |
